# Highly Branched Betulin Based Polyanhydrides for Self-Assembled Micellar Nanoparticles Formulation

**DOI:** 10.3390/ijms231911462

**Published:** 2022-09-28

**Authors:** Daria Niewolik, Barbara Bednarczyk-Cwynar, Piotr Ruszkowski, Grzegorz Dzido, Katarzyna Jaszcz

**Affiliations:** 1 Department of Physical Chemistry and Technology of Polymers, Silesian University of Technology, M. Strzody 9, 44-100 Gliwice, Poland; 2 Department of Organic Chemistry, Poznan University of Medical Science, Grunwaldzka 6, 60-780 Poznan, Poland; 3 Department of Pharmacology, Poznan University of Medical Science, Rokietnicka 3, 60-806 Poznan, Poland; 4 Department of Chemical Engineering and Process Design, Silesian University of Technology, M. Strzody 7, 44-100 Gliwice, Poland

**Keywords:** betulin, polyanhydrides, biodegradable polymers, cytostatic activity, polymeric micelles

## Abstract

Polyanhydrides based on betulin are promising materials for use in controlled drug delivery systems. Due to the broad biological activity of betulin derivatives and lack of toxicity in vitro and in vivo, these polymers can be used both as polymeric prodrug and as carriers of other biologically active compounds. In this study, we develop a novel amphiphilic branched polyanhydrides synthesized by the two-step melt polycondensation of betulin disuccinate (DBB) and a tricarboxylic derivative of poly(ethylene glycol) (PEG_COOH). DBB and PEG_COOH were used as the hydrophobic and hydrophilic segments, respectively. The content of DBB in copolymers was from 10 to 95 wt%. Copolymers were assessed for their cytostatic activity against various cancer cell lines. Compared to linear DBB and PEG-based polyanhydrides, the branched polyanhydrides exhibited higher anticancer activity. The obtained polymers were able to self-assemble in water to form micelles with hydrodynamic diameters from 144.8 to 561.8 nm. and are stable over a concentration range from 12.5 µg/mL to 6.8 mg/mL. The formed micelles were found to be spherical in shape using a scanning electron microscope. It was found that the structure and composition of polyanhydrides affected the hydrodynamic diameter of the micelles. The branched betulin-based polyanhydrides have the potential to serve as biodegradable polymer prodrugs or carriers for other bioactive compounds.

## 1. Introduction

In recent years, polymers have attracted growing attention in pharmaceutical and biomedical applications. Compared to non-biodegradable polymers, which can cause toxicity due to accumulation in the body, biodegradable polymers exhibit superiority properties. They are promising materials for the preparation of novel drug delivery systems, due to their biodegradability, biocompatibility, and minimal cytotoxicity [1]. Biodegradable polymers used for biomedical application need to undergo biodegradation to biocompatible or harmless products, and their degradation rate and mechanical properties should match their potential application [2]. Additionally, they must not provoke a constant inflammatory effect in the body. The possibility of a polymer itself to exhibit medicinal properties [1] is another potential benefit. Some polymers, which fit some of the criteria and thus can be used in medical industry, include polyesters, polyurethanes, poly(orthoesters), polysaccharides, polyanhydrides [3], poly(amino acids) and polypeptides [4]. Among those, polyanhydrides are the most suitable for use in controlled drug delivery systems.

Polyanhydrides are a promising class of polymers for controlled drug delivery due to their favorable properties, such as biodegradability, surface erosion and low to no toxicity [5]. They have a high reactivity with water, which results in a rapid hydrolytic degradation. Due to their tendency to degrade by surface erosion, they are excellent material for controlled release carriers, because the rate of drug release can be controlled by the rate of polymer degradation [6]. Polyanhydrides have been approved by the FDA (Food and Drug Administration) for human use, as the degradation products are easily removed from the body by human metabolism [7]. Polyanhydrides have been already investigated for the controlled release of drugs, such as antineoplastic agents, local anesthetics, anticoagulants, antibiotics, and many others, in the form of spherical carriers, e.g., microspheres, nanoparticles [1]. Other examples of using polyanhydrides in medicine are copolymers of sebacic acid and erucic acid dimer used for the manufacture of implants for use in the treatment of osteomyelitis [8] or copolymers of poly(ethylene glycol) and sebacic acid for ophthalmic delivery of dorzolamide [9]. In our recent studies, our team obtained linear polyanhydrides based on betulin disuccinate (DBB) [10] and their copolymers with dicarboxylic derivatives of poly(ethylene glycol) (PEG) [11] and sebacic acid [12]. Polymers based on betulin can be used as a polymeric prodrug, releasing DBB (with anticancer activity) during hydrolysis under physiological conditions or as carriers for other biologically active compounds. 

In addition to the polyanhydrides’ use in drug delivery, they have a new potential use case as medical electronic devices. In the literature, there are reports of water-activated biodegradable and biocompatible battery, where the electrodes are encapsulated within a polyanhydride shell [13]. Another report describes the use of polyanhydrides within biodegradable brain sensors, which enable wireless data collection in body cavities and in deep tissues with fully bioresorbable platforms [14]. These novel uses of polyanhydrides are only possible thanks to their surface-eroding properties.

Branched polymers are a class of polymers between linear polymers and polymer networks. They exhibit significantly different properties, such as high surface functionality, globular conformation, low intrinsic viscosities, and high solubilities, compared to linear polymers [15,16,17]. However, compared to linear polymers, the physicochemical properties of which are largely determined by the monomer repeat unit, the properties of branched polymers result from their end groups at the surface of the polymer and the topology of the polymer [18]. Branched polymers can be used both as therapeutic molecules carriers and as active compounds, i.e., as anti-inflammatory agents [19]. 

**Figure 1:**
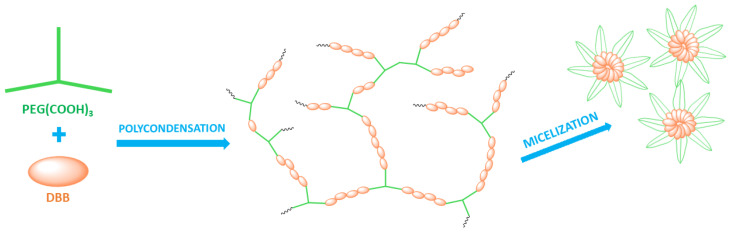
Scheme presenting the stages of the research.

The aim of our study was the synthesis and characterization of highly branched polyanhydrides based on betulin disuccinate and tricarboxylic derivatives of PEG and verifying the suitability of such polymers for micelles formation in self-assembling processes (Figure 1).

Linear poly(ethylene glycol) is often used in the synthesis of polymers for drug delivery due to its high aqueous solubility and the ability to influence the biodistribution in tumors through the enhanced permeability and retention (EPR) effect of parent drugs [20,21]. However, the linear PEG has only one or two functional groups, which can be used for reactions, thus limiting its use in the synthesis of branched and crosslinked polymers. Therefore, multiple functional PEG are preferable to us in the synthesis of branched polymers.

Previously, we described the synthesis and characterization of linear polyanhydride based on betulin disuccinate [10] and its linear copolymers [11,12]. Obtained polyanhydrides exhibit anticancer activity against cancer cell lines, such as HeLa, MCF-7, A-549, U-87MG, KB and HepG2, with the limited or lack of toxicity toward normal cells. In this study, a tricarboxylic derivative of PEG was selected as the branching agent. In our previous report, we describe the linear polyanhydrides, in which we used a linear dicarboxylic derivative of PEG (with molecular weights 250 or 600) as a comonomer [11]. The use of linear PEG increased the solubility and anticancer activity of copolymers. In this research, we wanted to assess the suitability of tricarboxylic PEG as the branching agent and how the architecture of polyanhydride can influence its chemical and biological activity.

The obtained branched betulin-based polyanhydrides were characterized by their structure, molecular weight and self-assembling properties. Betulin disuccinate was chosen as the hydrophobic segment, due to its biological activity, in the hope of obtaining polyanhydrides with potential anticancer activity. Trifunctional PEG was chosen as the hydrophilic segment, which was modified with succinic anhydride, to obtain derivatives with three carboxyl groups, which could subsequently serve as a branching agent in the polycondensation reaction. The obtained copolymers were tested for cytotoxicity against various cancer cell lines, including HeLa, U-87, KB, MCF and A-549. Betulin based branched polyanhydrides are able to self-assembly in water to form stable micelles in the wide range of concentration. Furthermore, the hydrodynamic diameters, zeta potential and shape of micelles were investigated by dynamic light scattering (DLS) and scanning electron microscope (SEM).

## 2. Results and Discussion

### 2.1. Preparation and Characterization of Tricarboxylic Derivative of Poly(Ethylene glycol) PEG(COOH)_3_

**Figure 2:**
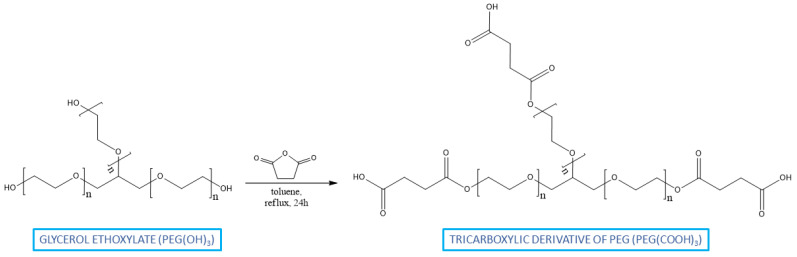
Reaction scheme for the modification of PEG.

A tricarboxylic derivative of poly(ethylene glycol) (PEG(COOH)_3_) was synthetized by the reaction of trifunctional PEG with succinic anhydride in reflux for 24 h. The reaction was carried out in toluene, with a 3-fold molar excess of succinic anhydride (Figure 2). 

**Figure 3:**
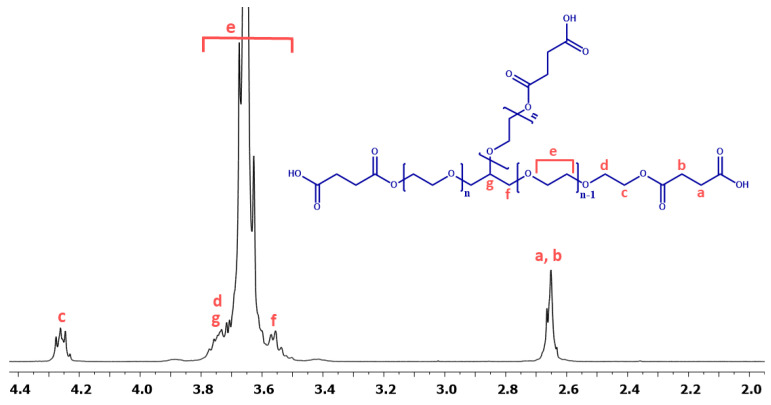
^1^H NMR and spectrum of PEC_COOH.

The purpose of the modification was to obtain a PEG derivative that could serve as a branching agent in the polycondensation reaction. PEG(COOH)_3_ was obtained in high yield (>90%), and its chemical structure was confirmed by NMR spectroscopy (Figure 3). 

The resulting derivative of PEG is a colorless viscous liquid, soluble in acetone, methylene chloride (DCM), chloroform and alcohols. Tricarboxylic derivatives of PEG were used as a branched agent for the betulin-based polyanhydrides synthesis. 

### 2.2. Synthesis and Characterization of Branched Betulin-Based Polyanhydrides

**Figure 4:**
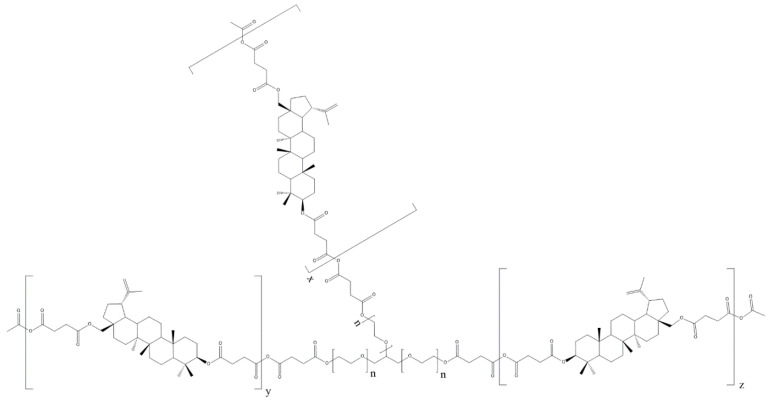
Structure of polyDBB_PEG_COOH.

A series of novel highly branched polyanhydrides were obtained by the two-step melt polycondensation of betulin disuccinate (DBB) and tricarboxylic derivatives of poly(ethylene glycol) (PEG(COOH)_3_) with the use of acetic anhydride (Figure S1). The content of PEG(COOH)_3_ in copolymers ranged from 5 to 90 wt%. The obtained polyanhydrides were solid and amorphous materials. The chemical structure of polyDBB_PEG_COOH shown in Figure 4 was confirmed by spectroscopic methods FT-IR, ^1^H NMR and ^13^C NMR. The presence of two characteristic bands at 1733 cm^−1^ and 1822 cm^−1^ in the carbonyl region of the FT-IR spectra (Figure S2) affirms that polyanhydrides have been obtained. Peaks at 1098 and 968 cm^−1^ correspond to the symmetric and asymmetric stretching modes of C-O-C for the segments of ethylene glycol. The peaks are described above, which indicate that the branched polyanhydrides contain both the anhydride and ethylene glycol segments.

**Figure 5:**
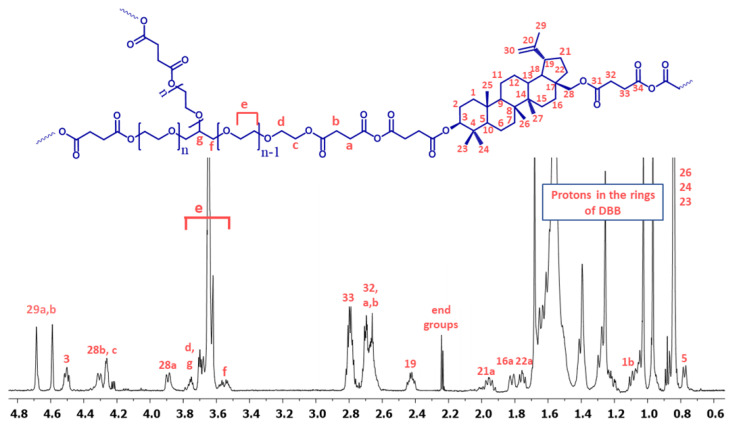
^1^H NMR spectrum of polyanhydrides based on betulin disuccinate and PEG(COOH)_3_.

Figure 5 shows the typical ^1^H NMR spectrum of the copolymers. In the ^1^H NMR spectra, the signal at δ = 2.84–2.74 ppm (C_33_-H_2_ and c) is visible, which could be assigned to methylene protons close to anhydride groups in the DBB and PEG part. The presence of this signal in the ^1^H NMR spectrum further confirmed the formation of the polyanhydride.

The ^13^C NMR spectra (Figure S3) of the copolymers showed signals assigned to carbonyl carbon atoms in anhydride (δ = 168.52 ppm) and ester groups (δ = 172.27 ppm and δ = 171.65 ppm) as well as two different signals of methylene carbon atoms next to ester (δ = 29.30 ppm and δ = 29.02 ppm; C_32_ in DBB and b in PEG) and anhydride (δ = 31.00–30.75 ppm; C_33_ in DBB and a in PEG) groups, respectively. 

In the ^1^H NMR and ^13^C NMR spectra, besides the signals confirming the presence of ester and anhydride bonds, signals attributed to methylene protons or methylene carbon atoms present in the repeating unit of PEG(COOH)_3_ were also observed. The presence of the signals at δ = 3.72–3.58 ppm (e) in ^1^H NMR and δ = 70.95 ppm in ^13^C NMR spectra confirms the presence of the repeating unit of PEG(COOH)_3_ in polyanhydrides. Additionally, signals attributed to other methylene protons (δ = 4.26 ppm, δ = 4.22 ppm and δ = 3.58–3.50 ppm) or methylene carbon atoms (δ = 71.26 ppm, δ = 69.30 ppm and δ = 64.50 ppm) in PEG were also observed. 

**Figure 6:**
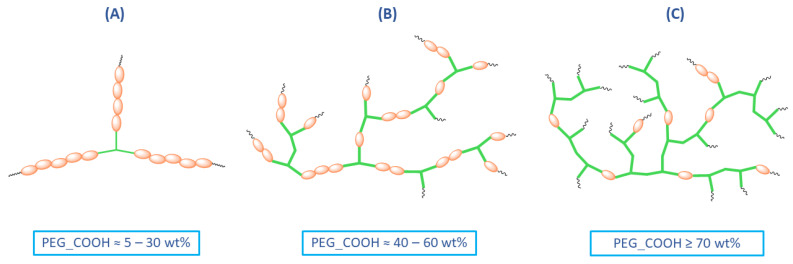
Three types of DBB—PEG(COOH)_3_ branched polyanhydrides: (**A**) structure of polyanhydrides containing 5–30 wt% of PEG_COOH; (**B**) structure of polyanhydrides containing 40–60 wt% of PEG_COOH; (**C**) structure of polyanhydrides containing ≥70 wt% of PEG_COOH.

The rest of the ^1^H NMR and ^13^C NMR signals, assigned to the relevant protons and carbons of repeating unit of DBB segments, have been discussed in detail in our previous work [10]. ^1^H NMR was used to determine the number of DBB molecules per one PEG molecule. The DBB to PEG_COOH ratio was calculated according to Equations (1)–(3). The intensities of signals used to calculate DBB to PEG_COOH ratio (Figure S4) in the obtained polyanhydrides are summarized in the Tables S1–S10. Most copolymers have higher weight ratios of DBB segments to PEG(COOH)_3_ segments than the feeding composition. Depending on the PEG_COOH content in copolymers, we can distinguish three hypothetical structures of the resulting branched polyanhydrides (Figure 6).

For polyanhydrides containing from 5 to 30 wt% of trifunctional PEG, the predicted structure had the shape of a three-pointed star, in which the PEG molecule was the core and the arms were composed of linked DBB (Figure 6A). The number of DBB molecules per one arm of the PEG was calculated based on NMR spectra and ranges from 10 (DBB_PEG_COOH_5) to 2 (DBB_PEG_COOH_30). As the PEG content increases (40–60 wt%), the structure of the copolymers became increasingly branched (Figure 6B), with 2–3 DBB molecules per one PEG (approximately one DBB per one arm of PEG). For copolymers containing above 70 wt% of PEG_COOH (Figure 6C), the polyanhydrides consist mostly of PEG molecules linked together (with approximately one DBB molecule per one PEG).

**Figure 7:**
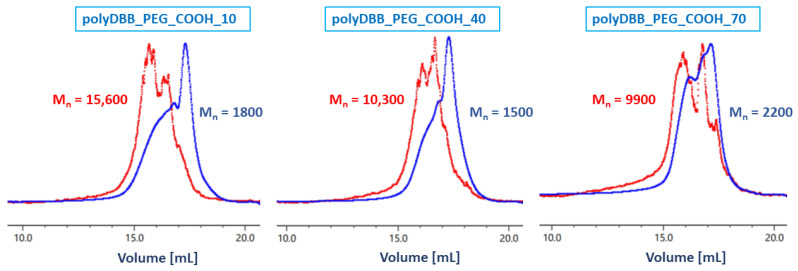
Comparison of chromatograms for copolymers containing 10, 40 and 70 wt% of PEG_COOH obtained from GPC (blue curve) and MALS (red curve).

The molecular weights of the branched polyanhydrides were determined by gel-permeation chromatography (GPC) and multiangle light scattering (MALS). The molecular weights of branched copolymers determined by GPC were much lower than the Mn determined by MALS and ranged from 1700 to 8000 g/mol. GPC measurements are based on polystyrene standards; thus, the obtained molecular weights of polyanhydrides are not real molecular weights but rather a relative comparison to polystyrene. Additionally, high molecular weight molecules can appear both at normal and larger than expected elution volumes due to the mechanical entanglement of branched molecules in the column packing and their later elution together with smaller molecules [22]. Figure 7 shows the differences in the appearance of the GPC and MALS chromatograms.

MALS is the better method for determining the molecular weights of branched polymers compared to GPC because it allows the determination of the actual molecular weights (Table 1). 

The molecular weights of copolymers determined by MALS ranged from 9900 to 16,000 g/mol. The highest average Mn was observed for polymers containing 10 and 20 wt% of PEG(COOH)_3_, in which the hydrolytic degradation is the slowest due to the lower hydrophilicity of the copolymers.

**Figure 8:**
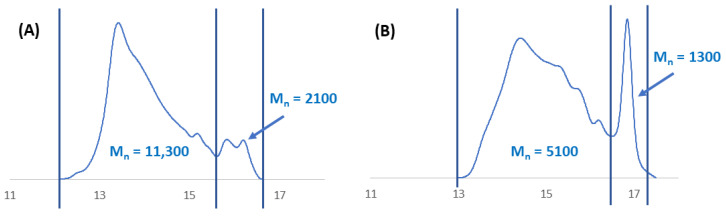
Gel-permeation chromatography (GPC) chromatograms of polyDBB_PEG_COOH_30 (**A**) and polyDBB_PEG_COOH_60 (**B**).

The molecular weights of polyanhydrides may be underestimated due to the partial degradation of the polymers during the measurement. Polyanhydrides hydrolytic instability may cause a decrease in the polymer molecular weight during the preparation of the solutions and measurements. The polyanhydrides could begin to partially degrade at the moment of dissolving in DCM, thus affecting the measurements. The GPC chromatogram of most polyanhydrides exhibited a bimodal character (Figure 8) and indicated the presence of low molecular fraction, which was likely due to the degradation products. This is evident by the presence of the signal that can be predominantly attributed to the PEG molecule.

The thermal properties of the polyanhydrides, presented in Table 1, were investigated using the DSC method. Obtained copolymers were completely amorphous, and no crystallinity was observed within the temperature range of −90 to 250 °C. As in the case of linear polyanhydrides, even the low PEG(COOH)_3_ content reduces the Tg compared to homopolymers (polyDBB, Tg = 124 °C). Copolymers containing up to 20 wt% of PEG retain Tg above room temperature. Polymers containing 40 wt% and more of PEG have Tg below 0 °C. Polyanhydride containing 30 wt% of PEG have two different glass temperature: one related to polyPEG(COOH)_3_ (Tg = −22.2 °C) and the second one related to polyDBB segments (Tg = 54.3 °C). 

Copolymers were found to be insoluble in water, ethanol (except for copolymers containing 5 and 10 wt% of PEG, which are partially soluble), diethyl ether and hexane, but were dissolvable in toluene, methylene chloride, chloroform and THF. Polymers containing 60 wt% and above of PEG were also soluble in DMSO. The results of the polyanhydrides solubility tests are summarized in the Table S11.

**Figure 9:**
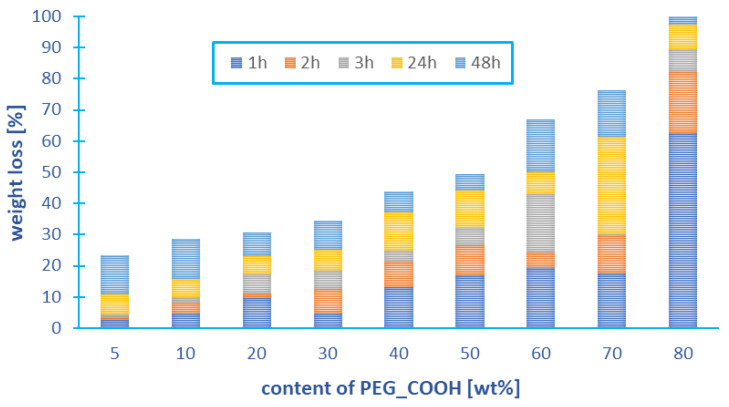
Weight loss of branched copolymers based on DBB and PEG_COOH during hydrolytic degradation in phosphate buffer conducted at 37 °C (Experiments were performed in triplicate).

Branched polyanhydrides based on DBB and PEG_COOH undergo hydrolytic degradation under physiological conditions (37 °C, pH = 7.4) to betulin disuccinate that exhibits anticancer activity and tricarboxylic derivative of PEG approved by the FDA. The hydrolytic degradation was monitored by recording the mass loss of the test samples, according to Equation (4). The results of the experiment are shown in Figure 9.

Under physiological conditions, branched polyanhydrides degrade completely in about 24 h. After this time, the complete disappearance of anhydride bonds was observed on the NMR spectra of the degradation samples. The weight loss of the tested samples was dependent on the PEG_COOH content in polyanhydrides. After 48 h, the weight loss of copolymers containing up to 30 wt% of PEG was below 40% (*w*/*w*), which was due to the poor solubility of the degradation product (betulin disuccinate) in PBS. The degradation rate of the polyanhydrides increases strongly with the increased PEG_COOG content in the polyanhydrides, which is due to their increased hydrophilicity. The highest weight loss was observed for the copolymer containing 80 wt% of PEG (100% (*w*/*w*)). 

Branched polyanhydrides were also studied to determine their cytostatic activity against selected cancer cell lines, including cervix, breast, lung, central nervous system and nasopharynx tumors. The cell lines were used to find the concentrations causing the inhibition of cell growth in culture by 50% (IC_50_). The IC_50_ values obtained for DBB, polyDBB and the copolymers are listed in Table 2. Cytarabine was used as the internal standard for methodological evaluation, and actinomycin D (widely used anticancer agent) was used as a point of reference to compare with our compounds and their activity. In Table 2, the results also include values obtained for monomer (DBB) and polyDBB, as described in an earlier study [10]. Cytostatic activity studies have not been conducted for the polyanhydride containing 90 wt% of PEG, which was due to its high hydrophilicity and rapid degradation.

The cytostatic tests indicated that the obtained copolymers were effective in the inhibition of growth of cancer cells (IC_50_ < 12 µg mL^−1^). The results obtained so far on the anticancer activity of polyanhydrides confirmed that the cytostatic activity is dependent on the amount of DBB and increases with the increase in DBB content in polyanhydrides. Additionally, the biological activity of the copolymer is dependent on the hydrolytic degradation rate and therefore on the PEG content. The polyanhydride containing 20 wt% of PEG(COOH)_3_ showed the highest cytostatic activity (IC_50_ values in the range of 3.08 to 3.88 μg mL^−1^, depending on the type of cancer cell lines). Both polyDBB and PEG(COOH)_3_ show a lack of cytotoxicity toward normal cells (IC_50_ = 27.13 and 59.04 μg mL^−1^, respectively), so polymers based on them should be safe for humans.

**Figure 10:**
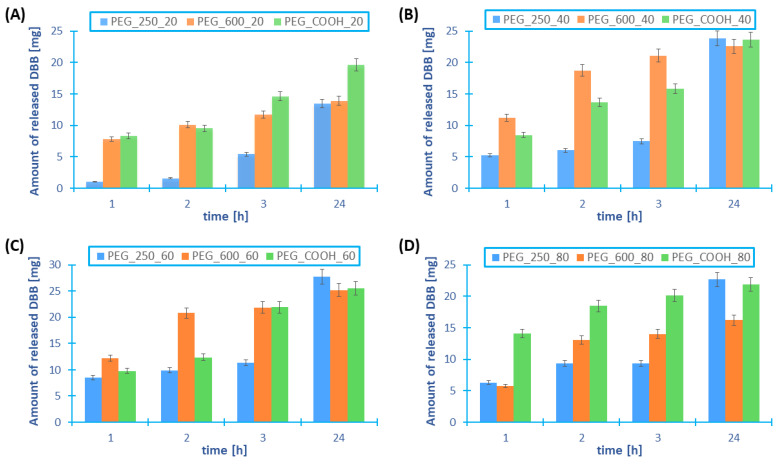
Comparison of the amount of DBB released from branched and linear copolymers, in which the PEG content is, respectively, 20 wt% (**A**), 40 wt% (**B**), 60 wt% (**C**), and 80 wt% (**D**) (*n* = 3, error bars, standard deviation).

In most cases, branched polyanhydrides exhibit greater cytostatic activity compared to linear polyanhydrides containing the same PEG content. This is due to the release of larger amounts of DBB during the degradation of branched copolymers than in the case of linear polyanhydrides. The results are summarized in Figure 10. 

From the obtained results, it can be concluded that the amount of DBB released from branched polyanhydrides is higher than the amount of DBB released from PEG 600-based linear polyanhydrides. However, in the case of linear copolymers containing PEG 250, the highest DBB release was observed for linear rather than branched polymers. The exception is the branched copolymer containing 20 wt% of PEG, for which the amount of released DBB is higher than in linear copolymers. Proof of the better utility of using branched polyanhydrides as prodrugs is the fact that the linear polyanhydrides based on DBB and PEG, containing more than 40 wt% of PEG, cannot be used in controlled drug release systems. This is due to their strong hydrophilicity causing their rapid degradation, making it impossible to obtain, for example, microspheres or polymer nanospheres, that can be used as a platform to deliver DBB or other biologically active compounds. Branched polyanhydrides also show high hydrophilicity, but their better stability and the possibility of obtaining micelles, which is impossible in the case of linear copolymers, allows them to be used in controlled drug delivery systems. 

To obtain polymeric prodrugs, the biologically active compounds can be attached to the polymer side chain [23,24] or, as in our case, incorporated into the polymer backbone. PolyDBB_PEG_COOH is a polymeric prodrug system in which the drug is chemically incorporated into the polymer backbone, with the actual active agent (DBB) becoming available as the polymer degrades. The anticancer activity of DBB-based polyanhydrides depends on the degree of hydrolytic degradation of the polymer and on the amount of DBB released. Thus, copolymers based on betulin disuccinate and PEG(COOH)_3_ can be used as degradation-based delivery systems for DBB, or, combined with other chemotherapeutic agents, they can lead to a synergistic therapeutic effect in cancer treatment.

The cytotoxicity of polymers is a complex assessment and is also affected by polymer shape and flexibility. Branched polymers offer an ability to modulate toxicity to cells [18]. Considering the low toxicity of betulin-based polyanhydrides, their cytostatic activity toward the cancer cell lines and the highly branched structures, these polymers look promising as a potential biomaterial and carriers for use in medicine.

### 2.3. Preparation and Characterization of Polymeric Micelles 

**Figure 11:**
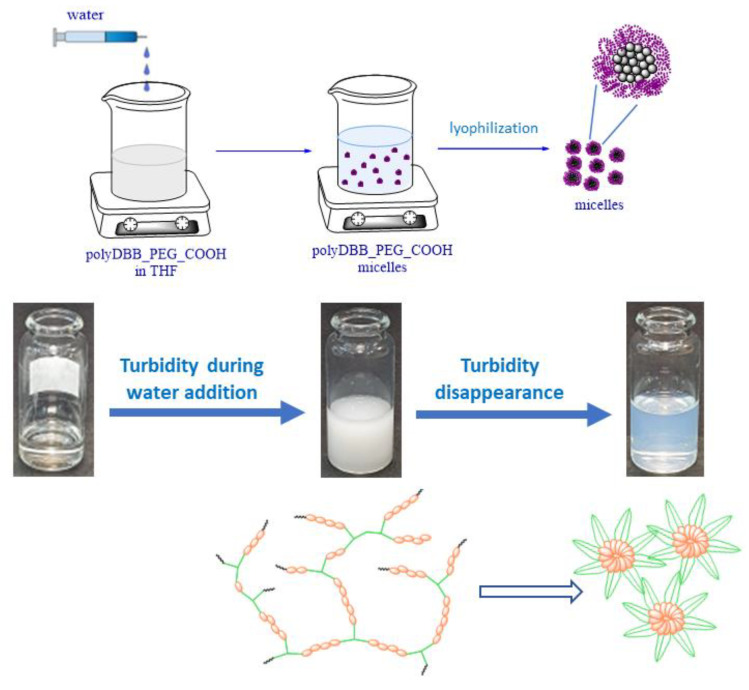
Scheme of micelle preparation.

The micelles from branched polyanhydrides based on DBB and PEG (polyDBB_PEG_COOH) were prepared by adding water via a syringe into a solution of polyDBB_PEG_COOH in tetrahydrofuran. THF, which is miscible with water, was used as the solvent for branched betulin-based polyanhydrides. The DBB part could constitute an internal hydrophobic core, while a tricarboxylic derivative of PEG could provide a hydrophilic outer shell of the micelles. After the water was dropped into the THF solution of copolymers, the THF phase was mixed with water immediately, and the copolymer was confronted with a more hydrophilic environment. Initially, the turbidity of the solution was observed under the influence of the added water, which was most probably due to polymer precipitation (because of the hydrophobicity of betulin disuccinate). However, as water was added further, the turbidity gradually disappeared until an opalescent solution was obtained (Figure 11). The observed changes indicate the formation of micellar structures.

For comparison, a similar experiment was carried out with the linear copolymers based on DBB and PEG (described in our previous article [11]), but in this case, only turbidity was observed, indicating the precipitation of the polymer under the influence of the added water. In the case of linear polyanhydrides based on DBB and linear PEG, no formation of micellar structures was observed.

**Figure 12:**
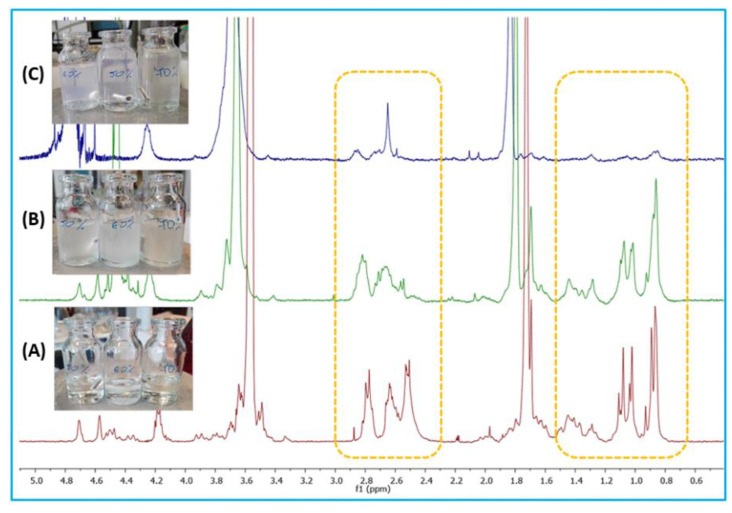
Micellization process observed by NMR spectroscopy: (**A**) solution of copolymer in THF; (**B**) appearance of turbidity and (**C**) opalescent solution.

The micellization process was also observed by ^1^H NMR spectroscopy. For this, copolymer was dissolved in deuterated THF and different D_2_O content was added to the polymer solution. For each branched copolymer, 4 samples of 0.6 ml of solution were prepared, to which appropriate amounts of deuterated water were then added, at which the beginning of turbidity, the maximum turbidity and its disappearance were observed. NMR spectra were recorded for the polymer solution in THF, at the moment of the appearance of turbidity, due to the addition of water and at the moment of the disappearance of turbidity (opalescent solution). Figure 12 present the recorded NMR spectra for copolymer containing 40 wt% of PEG. 

DBB is a hydrophobic compound, so when adding water, these segments will tend to hide inward to minimize contact with the water. In the ^1^H NMR spectra presented in Figure 12, it can be seen that with increasing content of D_2_O, the signals of protons belonging to DBB disappear. At the point at which turbidity of the solution disappeared, those signals became practically invisible. However, the signals belonging to the protons assigned to PEG did not change their intensity. This is indicative of the formation of a core-shell micellar system. Similar results were not observed for linear polyanhydrides, which excludes the possibility of micelles formation from them.

Micelles can be prepared from branched polyanhydrides containing 40–80 wt% of PEG(COOH)_3_ in the wide range of concentration. The maximum polymer concentration (6.85–7.63 mg/mL) at which micelles are stable was determined by two methods: transmittance measurement method (Figure S5) and pyrene fluorescence method (Figure S6). The results are presented in Table S12. Obtained micellar solutions can be diluted without loss of the stability to concentration 0.0125 g/mL. Stability was examined by determination of sizes and zeta potential of micelles for micellar solutions with various concentration (Table S13).

**Figure 13:**
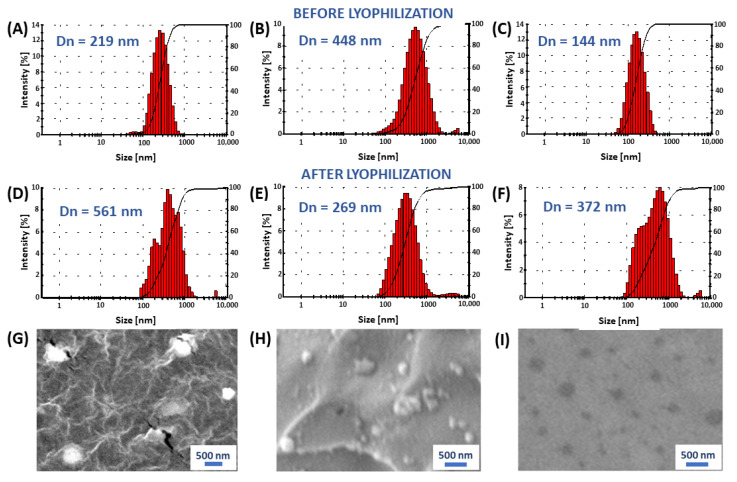
The size distribution of micelles before lyophilization: from polyDBB_PEG_COOH_40 (**A**), polyDBB_PEG_COOH_60 (**B**) polyDBB_PEG_COOH_80 (**C**); after lyophilization: from polyDBB_PEG_COOH_40 (**D**), polyDBB_PEG_COOH_60 (**E**), polyDBB_PEG_COOH_80 (**F**) and their morphologies after lyophilization (**G**–**I**) characterized by SEM.

In order to investigate the micellar solution of copolymers, the hydrodynamic diameters and polydispersity indices (PDI) of the polyanhydride micelles were measured by DLS. The results obtained by DLS before lyophilization (in the solution with maximum concentration) and after lyophilization (redispersed in the distilled water) are listed in Table 3. and presented in Figure 13.

The diameter of the branched copolymer micelles was found to increase with the increasing of the PEG content in polyanhydride (before lyophilization). For micelles obtained from copolymer containing 80 wt% of PEG(COOH)_3_ the hydrodynamic diameter is different. It is the smallest diameter compared to other copolymer micelles. The zeta potential (ZP) values measured for micelles after lyophilization were within the ranges from −15.5 to −31.3 mV and were slightly higher compared to the ZP values measured for micelles before lyophilization. Potential zeta was dependent on the concentration of micellar solutions and was the lowest for concentration 12.5 µg/mL (ZP = −47.8 mV). The obtained data indicate that micelles presented a negative surface change. 

Micellar structures observed on SEM images are spherical (Figure 13). The samples for SEM were prepared by dropping the micellar solution onto a carbon tape, after which the micellar solution was lyophilized. The size disparity between the micelles in SEM images and their original measured diameter is likely due to the fact that the SEM samples were applied in a dry state of micelles, leading to the shrinkage of micelles.

The diameter of the polyanhydride micelles depends on the structure of the copolymers, on the content of PEG in polymers, as well as the conditions of the preparation method. For copolymers containing 40 wt% of PEG, the hydrodynamic diameters of micelles after lyophilization are higher, which is due to their aggregation.

## 3. Materials and Methods

### 3.1. Materials

Betulin disuccinate (obtained in the laboratory according to the procedure described earlier [10]), succinic anhydride 99% (ACROS Organics, Geel, Belgium), toluene (STANLAB, Lublin, Poland), acetic anhydride (POCh S.A., Gliwice, Poland), glycerol ethoxylate (average M_n_~1000) (Sigma Aldrich, St. Louis, MO, USA), DMSO (Chempur, Piekary Śl., Poland), trichloroacetic acid (Chempur, Piekary Śl., Poland), acetic acid (Chempur, Piekary Śl., Poland), Tris buffer, sulforhodamine, tetrahydrofuran (Chempur, Piekary Śl., Poland), and methylene chloride (Chempur, Piekary Śl., Poland) were used as supplied. 

### 3.2. Cell Lines and Culture Conditions

KB, MCF-7 and HeLa cells were obtained from the European Collection of Cell Culture (ECACC) supplied by Sigma Aldrich. A-549 and U-87 MG cells were purchased from the American Type Culture Collection (ATCC) through LGC Standards (Lomianki). KB and U-87 cells were cultured in EMEM medium, while HeLa cells were grown in RPMI 1640 medium, A-549 cells were grown in F-12K medium and MCF-7 cells were grown in DMEM medium. Each medium was supplemented with 10% fetal bovine serum, 1% L- glutamine and 1% penicillin/streptomycin solution. All cultures were maintained at 37 °C in a humidified atmosphere containing 5% CO_2_.

### 3.3. Modification of Glycerol Ethoxylate (PEG(OH)_3_)

The modification of PEG(OH)_3_ was carried out as follows. First, 16.16 g of glycerol ethoxylate and 4.55 g of succinic anhydride were dissolved in 150 mL of toluene. The obtained solution was refluxed for 24 h under nitrogen atmosphere. After this time, the excess toluene was removed under vacuum. The obtained viscous liquid was obtained with a yield of over 90%. 

^1^H NMR (300 MHz, CDCl_3_, δ): 4.40–4.16 (m, -C(O)OCH_2_CH_2_O-), 3.96–3.36 (m, -CH_2_O- in repeating unit), 2,65 (s, -CH_2_C(O)OH I, -CH_2_C(O)O-).

^13^C NMR (75 MHz, CDCl_3_, δ): 172.13 (carbonyl), 78.38 (-CH-), 71.26 (-CH_2_-), 70.74-70.54 (most of carbon in repeating unit of PEG), 69.73 (-CH_2_-), 69.00 (-CH_2_-), 63.87 (-CH_2_), 29.30 (CH_2_COOH), 29.00 (CH_2_C(O)O).

### 3.4. Synthesis of Branched Polyanhydrides

Branched polyanhydrides were obtained by the two-step melt polycondensation of betulin disuccinate and tricarboxylic derivative of PEG (PEG_COOH), according to the procedure described earlier for linear polyanhydrides [11,12]. Betulin disuccinate and PEG_COOH mixed in defined ratios (Table 4) were refluxed in acetic anhydride (1:10, *w*/*v*) under nitrogen flow for 40 min. 

After this time, the excess of acetic anhydride and acetic acid formed in reaction was removed under vacuum. The remaining diacyl derivative of disuccinate betulin and PEG_COOH (prepolymer) was heated at 150 °C for 2 h with constant stirring under vacuum (0.1 mm Hg) and nitrogen. Copolymers (polyDBB_PEG_COOH) in the form of solid materials were obtained with a yield of over 90%. The obtained polymers were stored in a freezer.

FT-IR: v = 2941 cm^−1^ (m, v_C-H_), 2844 cm^−1^ (w, v_C-H_), 1822 cm^−1^ (m, v_C=O_), 1733 cm^−1^ (s, v_C=O_), 1034 cm^−1^ (v_C-O_). 

^1^H NMR (600 MHz, CDCl_3_, δ): 4.69 (1H, d, C_29_-H_a_); 4.59 (1H, d, C_29_-H_b_), 4.50 (1H, t, C_3_-H_α_); 4.30 (1H, d, C_28_-H_a_); 4.26 and 4.22 (c in PEG); 3.88 (1H, d, C_28_-H_b_); 3.80–3.72 (m, d and g in PEG); 3.72–3.58 (m, (O-CH_2_-CH_2_-) in PEG); 3.58–3.50 (m, f in PEG); 2.84–2.74 (4H, m, -OCOOC-CH_2_-CH_2_-COO-); 2.74–2.60 (4H, m, -OCOOC-CH_2_-CH_2_-COO-); 2.43 (1H, td, C_19_-H); 2.24 (end groups); 1.96 (1H, m, C_21_-H_a_); 1.82 (1H, d, C_16_-H_a_); 1.76 (1H, t, C_22_-H_a_); 1.68 (3H, s, C_30_-H_3_); 1.67–1.54 (7H, m, C_15_-H_a_, C_1_-H_a_, C_12_-H_a_, C_13_-H_a_, C_2_-H_a,b_, C_18_-H); 1.51(1H, m, C_6_-H_a_); 1.45–1.35 (5H, m, C_6_-H_b_, C_11_-H_a_, C_21_-H_b_, C_7_-H_a,b_); 1.29 (1H, d, C_9_-H); 1.26–1.17 (2H, m, C_11_-H_b_, C_16_-H_b_); 1.13–1.04 (3H, m, C_22_-H_b_, C_12_-H_b_, C_15_-H_b_); 1.02 (3H, s, C_25_-H_3_); 0.97 (3H, s, C_27_-H_3_); 0.94–0.92 (1H, m, C_1_-H_b_); 0.88–0.80 (9H, s, C_26_-H_3_, C_23_-H_3_, C_24_-H_3)_; 0.78 (1H, d, C_5_-H).

^13^C NMR (150 MHz, CDCl_3_, δ): 172.27 and 171.65 (C_q_, C(O)O); 168.52 (C_q_, C(O)OC(O)); 150.80 (C_q_, C-20); 109.99 (CH_2,_ C-29); 82.00 (CH, C-3); 78.38 (-CH- in PEG), 71.26 (CH_2_ in PEG); 70.95 ((O-CH_2_-CH_2_-) in PEG); 69.30 (CH_2_ in PEG); 64.50 (CH_2_ in PEG); 63.58 (CH_2,_ C-28); 55.76 (CH, C-5); 50.69 (CH, C-9); 49.24 (CH, C-18), 48.17 (CH, C-19); 46.91 (C_q_, C-17); 43.14 (C_q,_ C-14); 41.35 (C_q_, C-8); 38.77 (CH_2_, C-1); 38.25 (C_q_, C-10); 38.05 (CH, C-13); 37.49 (C_q_, C-4); 34.88 (CH_2_, C-22); 34.55 (CH_2_, C-7); 31.00–30.75 (CH_2_C(O)OC(O)); 30.12 (CH_2_, C-21); 30.03 (CH_2_, C-16); 29.30 and 29.02 (CH_2_C(O)O); 28.14 (CH_3_, C-23); 27.48 (CH_2_, C-15); 25.66 (CH_2_, C-12); 24.06 (CH_2_, C-2); 21.22 (CH_2_, C-11); 19.29 (CH_3_, C-30); 18.58 (CH_2_, C-6); 16.69 (CH_3_, C-25); 16.34 (CH_3_, C-26); 16.23 (CH_3_, C-24); 14.92 (CH_3_, C-27).

### 3.5. Characterization of Polymers

^1^H NMR and ^13^C NMR spectra of polymers and PEG(COOH)_3_ were recorded on a Varian 600 MHz spectrometer using CDCl_3_ (for PEG) and CD_2_Cl_2_ (for polymers) as the solvent and TMS as an internal standard. The DBB to PEG_COOH ratio was calculated from the ^1^H NMR spectra, based on Equations (1)–(3).

(1) $$I_{\left[1H\right]DBB}=\left(I_{C29-Ha}+I_{C29-Hb}+I_{C3-H}\right)/3$$

(2) $$I_{\left[1H\right]PEG\_COOH\;}=\left(I_{\delta =4.26\;ppm}+I_{\delta =4.22\;ppm}\right)/6$$

(3) $$\frac{DBB}{PEG\_COOH}=\frac{I_{\left[1H\right]DBB}}{I_{\left[1H\right]PEG\_COOH}}$$

where *I_[*1*H]DBB_* —intensity of one DBB proton, *I_[*1*H]PEG_COOH_*_—_intensity of one PEG_COOH proton, *I_(C*29*-Ha)_* and *I_(C*29*-Hb)_*—intensity of signal assigned to methylene protons at the double-bonded carbon (*δ* = 4.69 and 4.59 ppm), *I_(C*3*-H)_*—intensity of signal assigned to metine proton in the ring of betulin (*δ* = 4.50 ppm), *I_*(*δ=*4.26 ppm)*_* and *I_*(*δ=*4.22 ppm)*_*—intensity of signal assigned to methylene protons that are close to ester group in PEG.

Infrared (FT-IR) spectra were recorded by means of a PerkinElmer Spectrum Spectrometer. Spectra were recorded at 16 scans per spectrum in the range of 4000–400 cm^−1^ with a resolution of 1 cm^−1^. Molecular weights of polyanhydrides were determined in methylene chloride by gel-permeation chromatography (GPC) using an Agilent Technologies Infinity 1260 chromatograph that was equipped with a refractive index detector and calibrated with linear polystyrene standards (580–300,000 g/mol) and size exclusion chromatography (SEC) using an Agilent Pump 1100 Series equipped with a set of two PLGel 5 μm MIXED-C columns. A Wyatt Optilab Rex differential refractometer and a Dawn Eos (Wyatt Technology Corporation) laser photometer (MALS) were used as detectors. Thermal analyses were performed using a 822^e^ DSC Mettler Toledo differential scanning calorimeter. Samples of about 3–4 mg were tested in temperature range from −90 to 250 °C at a heating rate of 10 °C/min.

### 3.6. Hydrolytic Degradation of Copolymers

The hydrolytic degradations of branched polyanhydrides were performed in a phosphate buffer solution of pH 7.4 (PBS) at 37 °C. The hydrolytic degradation was monitored by recording the mass loss of the test samples. The solid samples of the copolymers (approximately 0.1 g) were placed in the weighed filters. Subsequently, the filters with copolymers were placed in glass vials containing 15 mL of PBS. The vials were incubated at 37 °C for a defined period of time (from 1 to 48 h). After incubation, the buffer solutions were decanted. The remaining samples were rinsed with distilled water, dried to constant weight in a vacuum oven, and weighted to the nearest 0.0001 g. The mass loss was defined as follows:

(4) $$\Delta m=\frac{m_{1}-m_{2}}{m_{1}}\times 100\%$$

where *m*_1_ represents the weight of the dry sample before degradation and *m*_2_ represents the weight of the dry sample after degradation at the defined time interval.

### 3.7. Cytostatic Activity of Polyanhydrides

The protein-staining sulforhodamine B (SRB) assay, developed by the National Cancer Institute (USA) for in vitro antitumor screening, was employed in this study for determination of the cytotoxic activity of test compounds. The SRB assay estimates cell densities based on the measurement of cellular protein content.

For the SRB assay, 100 µL of diluted cell suspension containing approximately 10^4^ cells was added to the wells of 96-well plates. After 24 h, when a partial monolayer was formed, the supernatant was aspirated, and 100 µL medium containing test compounds (copolymers) at six different concentrations (0.1, 0.2, 1, 2, 10 and 20 µg/mL) was added to the cells. Stock solutions of test compounds were prepared in DMSO, and the concentration of DMSO in the assay did not exceed 0.1%, which was found to be nontoxic to applied cell lines. After incubation for 72 h, 25 µL of 50% trichloroacetic acid was added to each well, and the plates were incubated for 1 h at 4 °C. After that, the plates were washed with distilled water to remove traces of medium and were air-dried. Then, the dried plates were stained with 100 µL 0.4% SRB (prepared in 1% acetic acid) for 30 min at room temperature. Unbound dye was removed by rapid washing with 1% acetic acid, and the plates were air-dried overnight. Finally, the protein-bound dye was dissolved in 100 µL of 10 mM unbuffered Tris, and the absorbance was read at 490 nm.

### 3.8. Formulation of Micelles

The micelles fabricated from branched polyanhydrides based on DBB and PEG (polyDBB_PEG_COOH) were prepared by adding water via a syringe into a solution of polyDBB_PEG_COOH in tetrahydrofuran (12.5 mg/mL). The amount of added water depended on the PEG_COOH content in polyanhydride. When water was added, the turbidity first appeared, which then gradually disappeared until an opalescent solution was obtained. The observed changes indicate the formation of micellar structures. After that, the micelles were lyophilized and stored in a freezer.

### 3.9. Maximum Polymer Concentration at Which Micelles Are Stable

The maximum polymer concentration was determined by two methods: (1) transmittance measurement method and (2) pyrene fluorescence method. Method I: First, 20.0 mg of copolymer was dissolved in 2 mL of toluene. Next, deionized water was added via syringe to the polymer solution mixed with a magnetic stirrer. Transmittance was measured every 0.5 mL of added water at the wavelength of 500 nm with a UV-Vis spectrophotometer (HITACHI-U-2910) at room temperature. Method II: Pyrene dissolved in THF was added to 5 mL vials (20 vials for each copolymer) to achieve, after adding polymer solution and water, a final concentration of 3 × 10^−4^ mol/dm^3^. THF was then evaporated, and 1 mL of polymer solution in THF (12.5 mg/mL) was added to vials. Next, while stirring, distilled water was added dropwise to samples in the amount of 0.1 to 2 mL, every 0.1 mL. For each copolymer, the concentration range of polymer solution in vials was from 4.2 to 11.4 mg/mL. The fluorescence excitation spectra were recorded on a Camlin fluoroSENS Pro spectrophotometer. The emission wavelength was carried out at 390 nm, and the excitation spectra were recorded from 300 to 420 nm. The ratio of the fluorescence intensity at 319 and 325 nm (I_319_/I_325_) was calculated and plotted against log concentrations of copolymers to determine the maximum polymer concentration at which micelles are still stable.

### 3.10. Characterization of Polymers and Micelles

The morphological characteristics of polymer micelles were carried out using a Phenom ProX scanning electron microscope (SEM) using an accelerating voltage of 10 kV. Samples were coated with a 5 nm gold layer. The average particle size and polydispersity (PDI) of micelles were determined by dynamic light scattering (DLS) using a Zetasizer Nano S90 (Malvern, England). Zeta potential (ZP) measurements of micelles were carried out using the Zetasizer S90 (Malvern, England). Before measurements, particles were dispersed in distilled water. Measurements were conducted at a pre-set temperature of 25 °C, which was reached after thermostating. The zeta potential was determined five times for each sample, with the final value being the arithmetic mean of the readings. ^1^H NMR spectra were recorded on a UNITY INOVA 300 MHz using D_2_O and deuterated THF as the solvent to confirm the formation of micellar systems.

## 4. Conclusions

Highly branched polyanhydride based on betulin disuccinate and tricarboxylic derivative of poly(ethylene glycol) with different DBB content were successfully synthetized. Polyanhydrides exhibit cytostatic activity toward cancer cell lines, such as HeLa, MCF-7, A-549, U-87 MG and KB. The obtained results for DBB, polyDBB and PEG(COOH)_3_ indicate the lack of toxicity toward normal cells, offering a promising point for further research into the copolymers’ use in medicine. This is further underlined by the existing FDA approval of carboxylic derivatives of PEG for use in drug delivery systems.

The polyDBB_PEG_COOH was found to be able to self-assemble to form micelles by simply adding water to the polymer solution in THF. DBB was used as a hydrophobic segment, while PEG was used as a hydrophilic segment. Micelles can be obtained from branched polyanhydrides containing 40–80 wt% of PEG. The diameters of the polymeric micelles were dependent on the copolymer structure and content of PEG in the polyanhydrides. PolyDBB_PEG_COOH micelles can be formed in the wide range of concentration (from 12.5 µg/mL to ~6.8 mg/mL), thereby obtaining a micellar solution that can be diluted to concentrations appropriate for administration. This is the first example of highly branched and biodegradable betulin-based polyanhydrides expected to have potential medical applications in drug delivery systems in the form of micelles.

## Figures and Tables

**Table 1:** Characteristic of branched polyanhydrides.

Polyanhydride	Number of DBB Molecules per 1 PEG Molecule (Feed Ratio)	Number of DBB Molecules per 1 PEG Molecule in Copolymers (^1^H NMR)	Molecular Weight (MALS)			DSC
			M_n_	M_w_	DP	T_g_ (°C)
DBB_PEG_COOH_5	39.9	30.8	13,400	49,800	3.72	103.4
DBB_PEG_COOH_10	18.9	13.3	15,600	51,600	3.32	64.0
DBB_PEG_COOH_20	8.4	10.8	16,000	48,800	3.05	78.6
DBB_PEG_COOH_30	4.9	6.9	12,100	33,300	2.75	−22.2; 54.3
DBB_PEG_COOH_40	3.2	3.5	9900	22,800	2.30	−31.2 −35.3
DBB_PEG_COOH_50	2.1	2.4	11,300	15,500	1.37	
DBB_PEG_COOH_60	1.4	2.0	10,900	14,700	1.35	−34.2
DBB_PEG_COOH_70	0.9	1.0	10,300	13,600	1.32	−40.7
DBB_PEG_COOH_80	0.5	0.6	12,500	16,400	1.31	−41.8
DBB_PEG_COOH_90	0.2	0.04	—	—	—	−43.2

DBB:PEG_COOH in polymer (^1^H NMR) calculated according to Equations (1)–(3), Tg—glass temperature of polymers determined as a midpoint of glass transition.

**Table 2:** Cytostatic activity of polyanhydrides against various cancer cell lines, expressed as IC_50_^a^.

Compound	Cytostatic Activity IC_50_ [µg/mL]				
	HeLa	U-87 MG	KB	MCF-7	A549
DBB ^b^	8.25 ± 0.81	7.37 ± 0.26	7.17 ± 0.93	7.25 ± 0.79	7.09 ± 0.01
polyDBB ^b^	16.23 ± 0.72	16.07 ± 0.02	17.81 ± 0.03	13.38 ± 0.06	16.19 ± 0.31
DBB_PEG_COOH_5	4.99 ± 0.06	4.08 ± 0.03	4.11 ± 0.01	4.51 ± 0.03	4.12 ± 0.01
DBB_ PEG _COOH_10	4.92 ± 0.04	4.16 ± 0.08	4.48 ± 0.02	4.05 ± 0.22	4.72 ± 0.03
DBB_ PEG _COOH_20	3.88 ± 0.01	3.87 ± 0.01	3.08 ± 0.07	3.82 ± 0.05	3.84 ± 0.01
DBB_ PEG _COOH_30	6.04 ± 0.08	6.77 ± 0.04	6.93 ± 0.03	6.04 ± 0.11	6.31 ± 0.03
DBB_ PEG _COOH_40	4.97 ± 0.04	5.13 ± 0.02	5.59 ± 0.01	5.94 ± 0.07	5.09 ± 0.02
DBB_ PEG _COOH_50	6.99 ± 0.06	6.74 ± 0.01	6.29 ± 0.01	6.88 ± 0.09	6.26 ± 0.03
DBB_ PEG _COOH_60	8.04 ± 0.05	8.11 ± 0.07	8.74 ± 0.02	8.05 ± 0.01	8.75 ± 0.12
DBB_ PEG _COOH_70	8.73 ± 0.02	8.28 ± 0.03	8.14 ± 0.12	8.09 ± 0.01	8.44 ± 0.09
DBB_ PEG _COOH_80	12.08 ± 0.06	12.77 ± 0.11	12.43 ± 0.17	12.99 ± 0.03	12.41 ± 0.03
PEG_COOH	36.07 ± 0.11	36.94 ± 0.93	36.12 ± 0.19	-	36.56 ± 0.76
Cytarabine ^c^	1.40 ± 0.08	1.03 ± 0.25	0.95 ± 0.02	-	1.17 ± 0.21

^a^ N = 3 (*t*-test), *p* < 0.05. ^b^ results from our previous work ^c^ Cytarabine and actinomycin D were used as the standard.

**Table 3:** The sizes of polyDBB_PEG_COOH micelles determined at maximum polymer concentration.

Polymer	Before Lyophilization		After Lyophilization	
	Dn ± SD [nm]	PDI	Dn ± SD [nm]	PDI
DBB_PEG_COOH_40	219.7 ± 3.3	0.213	561.8 ± 41.3	0.447
DBB_PEG_COOH_50	297.7 ± 2.7	0.229	295.6 ± 9.8	0.344
DBB_PEG_COOH_60	488.0 ± 17.0	0.356	269.4 ± 5.8	0.262
DBB_PEG_COOH_70	444.8 ± 9.1	0.272	290.0 ± 4.9	0.262
DBB_PEG_COOH_80	144.8 ± 0.9	0.150	372.2 ± 7.1	0.312

**Table 4:** Feed ratio of DBB and PEG_COOH.

Polyanhydride	Feed Ratio (% *w*/*w*)		Feed Ratio DBB:PEG_COOH (mol/mol)
	DBB	PEG_COOH	
polyDBB_PEG_COOH_5	95	5	1:0.02
polyDBB_PEG_COOH_10	90	10	1:0.05
0polyDBB_PEG_COOH_20	80	20	1:0.12
polyDBB_PEG_COOH_30	70	30	1:0.20
polyDBB_PEG_COOH_40	60	40	1:0.32
polyDBB_PEG_COOH_50	50	50	1:0.48
polyDBB_PEG_COOH_60	40	60	1:0.71
polyDBB_PEG_COOH_70	30	70	1:1.11
polyDBB_PEG_COOH_80	20	80	1:1.90
polyDBB_PEG_COOH_90	10	90	1:4.29
polyPEG_COOH	0	100	—

## Data Availability

The data presented in this study are available on request from the corresponding author.

## Supplementary Materials

The supporting information can be downloaded at: https://www.mdpi.com/article/10.3390/ijms231911462/s1.
