# Supplementary material for: Highly Branched Betulin Based Polyanhydrides for Self-Assembled Micellar Nanoparticles Formulation
Source: Int J Mol Sci. 2022 Sep 28;23(19):11462. doi: 10.3390/ijms231911462 (PMC9570305; doi:10.3390/ijms231911462)
Supplement: Supplementary file 1 [file ijms-23-11462-s001.zip › ijms-1847021-supplementary.pdf]

Supplementary materials

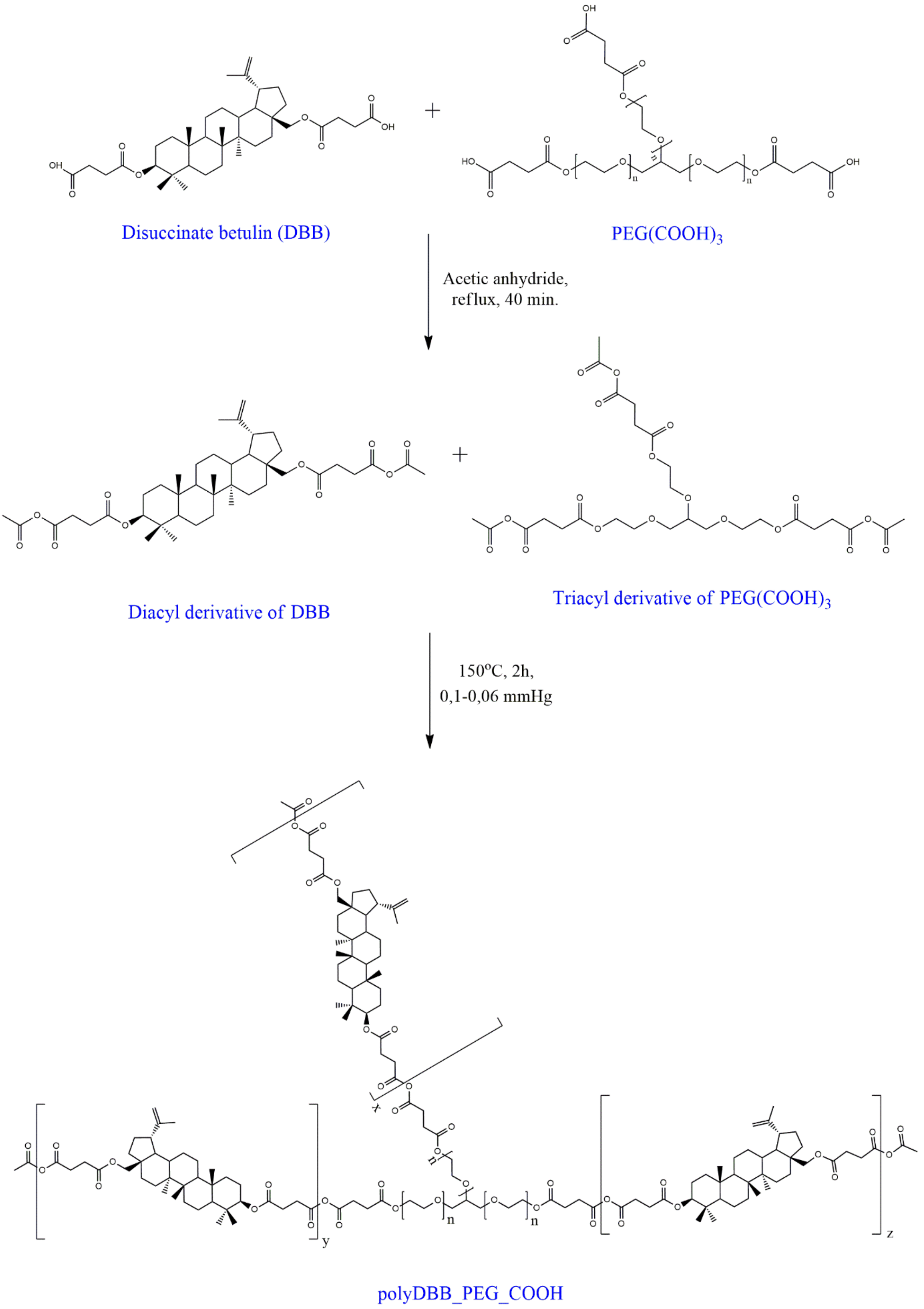

Figure S1. Reaction scheme of the synthesis of polyanhydrides based on disuccinate betulin (DBB) and tricarboxylic derivatives of poly(ethylene glycol).

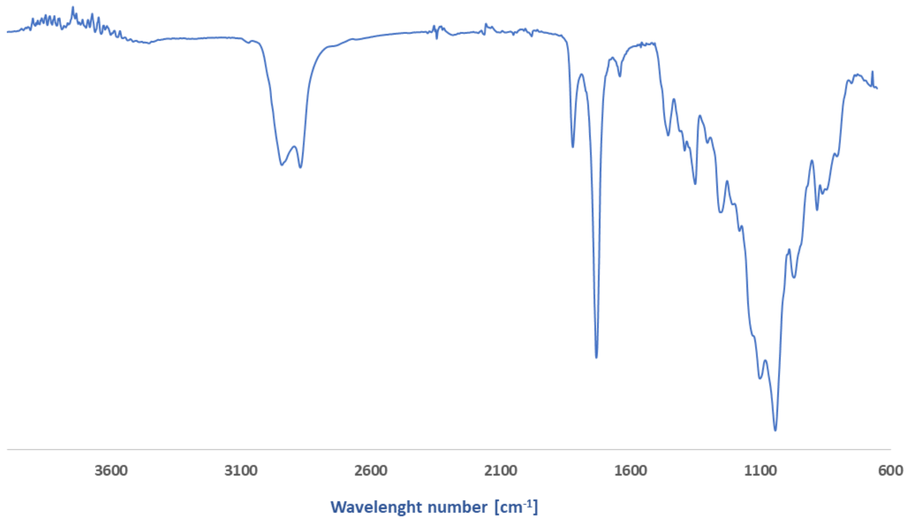

Figure S2. FT-IR spectrum of polyDBB_PEG_COOH.

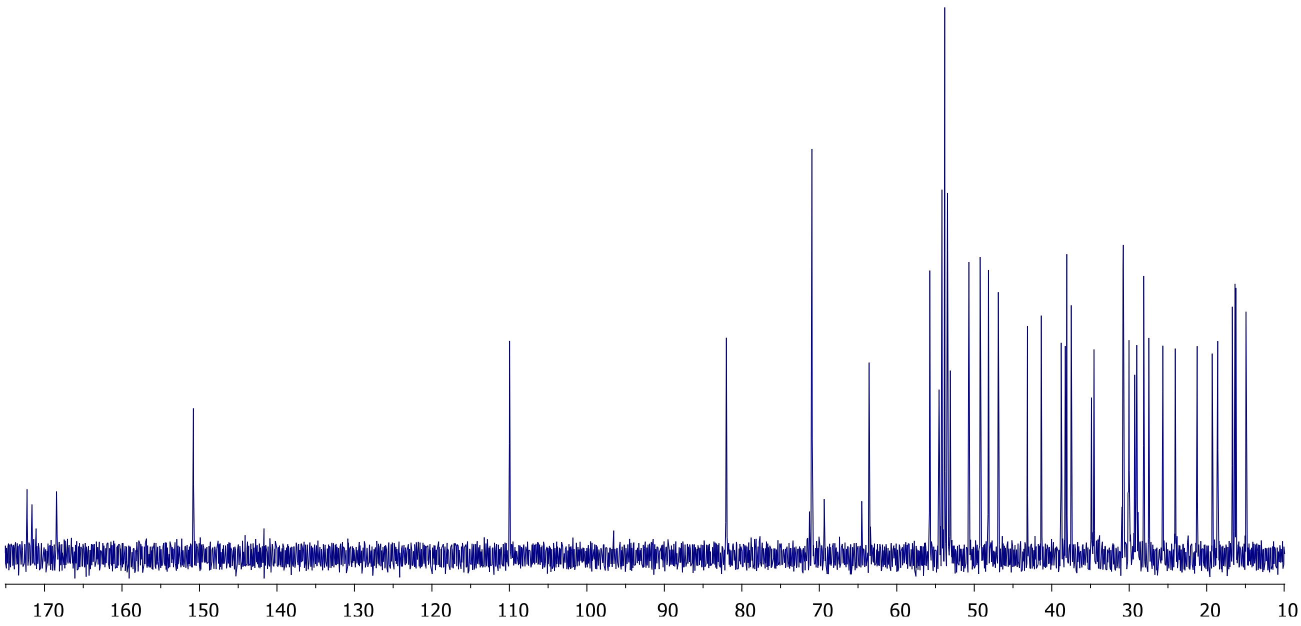

Figure S3. ^{13}C NMR spectrum of polyDBB_PEG_COOH.

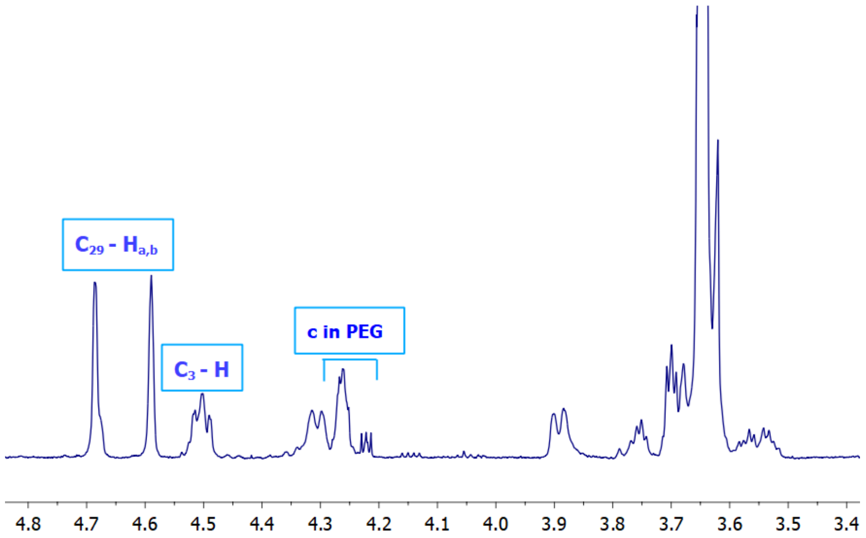

Figure S4. Fragment of the ^{1}H NMR spectrum of polyDBB_PEG_COOH.

Intensities of signals used to calculate DBB to PEG_COOH ratio in obtained polyanhydrides:

Table S1. Intensities of signals used to calculate DBB to PEG_COOH ratio in DBB_PEG_COOH_5.

Signal	Chemical shift δ [ppm]	Intensity	Assigned protons
C_3-H	4.50	1.52	~**CH**(-OCO)~
C_{29}-H_a	4.69	1.54	**H_2C**=C(-CH_3)-CH~
C_{29}-H_b	4.59	1.56	
c	4.26 and 4.22	0.33	-**CH_2**-O-C(O)-

Table S2. Intensities of signals used to calculate DBB to PEG_COOH ratio in DBB_ PEG_COOH_10.

Signal	Chemical shift δ [ppm]	Intensity	Assigned protons
C_3-H	4.50	1.81	~**CH**(-OCO)~
C_{29}-H_a	4.69	1.65	**H_2C**=C(-CH_3)-CH~
C_{29}-H_b	4.59	1.73	
c	4.26 and 4.22	0.81	-**CH_2**-O-C(O)-

Table S3. Intensities of signals used to calculate DBB to PEG_COOH ratio in DBB_PEG_COOH_20.

Signal	Chemical shift δ [ppm]	Intensity	Assigned protons
C_3-H	4.50	2.19	~**CH**(-OCO)~
C_{29}-H_a	4.69	1.55	**H_2C**=C(-CH_3)-CH~
C_{29}-H_b	4.59	1.87	
c	4.26 and 4.22	1.00	-**CH_2**-O-C(O)-

Table S4. Intensities of signals used to calculate DBB to PEG_COOH ratio in DBB_PEG_COOH_30.

Signal	Chemical shift δ [ppm]	Intensity	Assigned protons
C_3-H	4.50	2.28	~**CH**(-OCO)~
C_{29}-H_a	4.69	1.24	**H_2C**=C(-CH_3)-CH~
C_{29}-H_b	4.59	1.66	
c	4.26 and 4.22	1.44	-**CH_2**-O-C(O)-

Table S5. Intensities of signals used to calculate DBB to PEG_COOH ratio in DBB_PEG_COOH_40.

Signal	Chemical shift δ [ppm]	Intensity	Assigned protons
C_3-H	4.50	1.20	~**CH**(-OCO)~
C_{29}-H_a	4.69	1.06	**H_2C**=C(-CH_3)-CH~
C_{29}-H_b	4.59	1.13	
c	4.26 and 4.22	1.94	-**CH_2**-O-C(O)-

Table S6. Intensities of signals used to calculate DBB to PEG_COOH ratio in DBB_PEG_COOH_50.

Signal	Chemical shift δ [ppm]	Intensity	Assigned protons

C_3-H	4.50	1.09	~**CH**(-OCO)~
C_{29}-H_a	4.69	1.15	**H₂C**=C(-CH₃)-CH~
C_{29}-H_b	4.59	1.12	
c	4.26 and 4.22	2.76	-**CH₂**-O-C(O)-

Table S7. Intensities of signals used to calculate DBB to PEG_COOH ratio in DBB_PEG_COOH_60.

Signal	Chemical shift δ [ppm]	Intensity	Assigned protons
C_3-H	4.50	1.50	~**CH**(-OCO)~
C_{29}-H_a	4.69	0.98	$\mathbf{H_2C}$=C(-CH_3)-CH~
C_{29}-H_b	4.59	1.24	
c	4.26 and 4.22	3.73	-$\mathbf{CH_2}$-O-C(O)-

Table S8. Intensities of signals used to calculate DBB to PEG_COOH ratio in DBB_PEG_COOH_70.

Signal	Chemical shift δ [ppm]	Intensity	Assigned protons
C_3-H	4.50	0.68	~**CH**(-OCO)~
C_{29}-H_a	4.69	0.74	$\mathbf{H_2C}$=C(-CH_3)-CH~
C_{29}-H_b	4.59	0.62	
c	4.26 and 4.22	4.20	-$\mathbf{CH_2}$-O-C(O)-

Table S9. Intensities of signals used to calculate DBB to PEG_COOH ratio in DBB_PEG_COOH_80.

Signal	Chemical shift δ [ppm]	Intensity	Assigned protons
C_3-H	4.50	0.54	~**CH**(-OCO)~
C_{29}-H_a	4.69	0.42	$\mathbf{H_2C}$=C(-CH_3)-CH~
C_{29}-H_b	4.59	0.48	
c	4.26 and 4.22	4.61	-$\mathbf{CH_2}$-O-C(O)-

Table S10. Intensities of signals used to calculate DBB to PEG_COOH ratio in DBB_PEG_COOH_90.

Signal	Chemical shift δ [ppm]	Intensity	Assigned protons
C_3-H	4.50	0.06	~**CH**(-OCO)~
C_{29}-H_a	4.69	0.02	$\mathbf{H_2C}$=C(-CH_3)-CH~
C_{29}-H_b	4.59	0.04	
c	4.26 and 4.22	5.76	-$\mathbf{CH_2}$-O-C(O)-

Table S11. Solubilities of polyanhydrides.

Polyanhydride	Acetone	H_2O	EtOH	Toluene	Diethyl ether	THF	DMSO	$CHCl_3$	CH_2Cl_2	Hexane
polyDBB	—	—	—	+	—	+	±	+	+	—
DBB_PEG_COOH_5	+	—	±	+	—	+	—	+	+	—
DBB_ PEG _COOH_10	±	—	±	+	—	+	—	+	+	—
DBB_ PEG _COOH_20	±	—	—	+	—	+	—	+	+	—

DBB_ PEG _COOH_30	—	—	—	+	—	+	—	+	+	—
DBB_ PEG _COOH_40	—	—	—	+	—	+	—	+	+	—
DBB_ PEG _COOH_50	—	—	—	+	—	+	—	+	+	—
DBB_ PEG _COOH_60	—	—	—	+	—	+	+	+	+	—
DBB_ PEG _COOH_70	—	—	—	+	—	+	+	+	+	—
DBB_ PEG _COOH_80	—	—	—	±	—	+	+	+	+	—
DBB_ PEG _COOH_90	—	—	—	±	—	+	+	+	+	—
PEG_COO H	—	—	—	±	—	+	+	+	+	—

+ soluble, ± partially soluble, — insoluble.

Table S12. Maximum polymer concentration at which micelles are still stable.

Polymer	Maximum polymer concentration [mg/ml]	
	UV-Vis	Fluorimetry
DBB_PEG_COOH_40	6.85	7.19
DBB_PEG_COOH_50	7.12	7.29
DBB_PEG_COOH_60	7.06	7.30
DBB_PEG_COOH_70	—	7.63
DBB_PEG_COOH_80	—	—

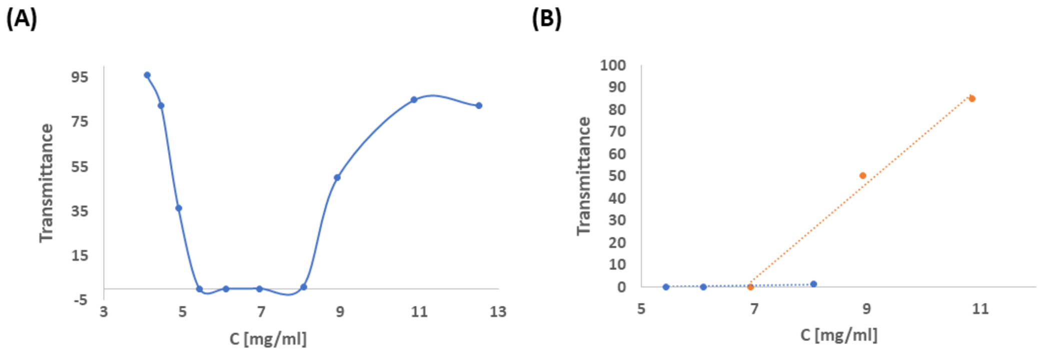

Figure S5. Dependence of transmittance on polymer concentration at a wavelength λ = 500 nm (A) and determination of maximum polymer concentration from transmittance versus concentration plot (B).

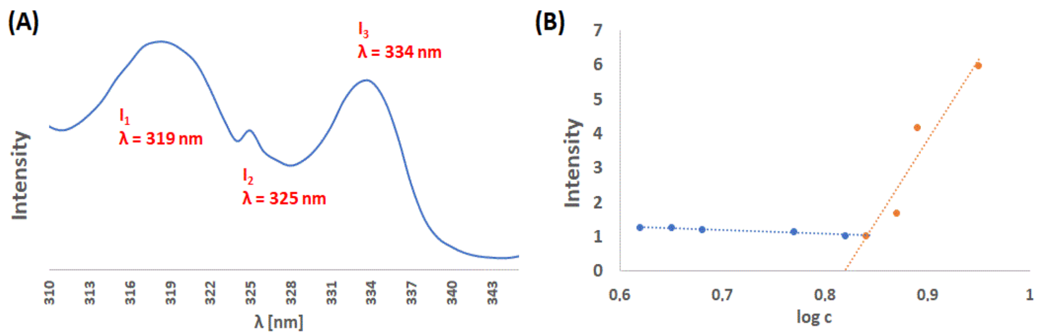

Figure S6. Excitation spectrum of pyrene in copolymer solution (A) and plots of intensity ratios I_{319}/I_{325} from pyrene excitation spectra versus log concentration of polyDBB_PEG_COOH (B).

Table S13. The sizes of micelles for the selected branched copolymers at three different concentrations.

Polymer concentration [mg/ml]	DBB_PEG_COOH_40		DBB_PEG_COOH_60		DBB_PEG_COOH_70	
	Dn ± SD [nm]	PDI	Dn ± SD [nm]	PDI	Dn ± SD [nm]	PDI
1.25	55.09 ± 0.13	0.172	250.7 ± 38.0	0.250	369.1 ± 1.7	0.048
0.125	48.65 ± 0.9	0.207	216.6 ± 2.8	0.271	294.3 ± 3.3	0. 091
0.0125	56.67 ± 1.6	0.493	227.8 ± 7.2	0.260	307.6 ± 0.7	0.131
